# Association Between Rest–Activity Rhythm and 27-Hydroxycholesterol (27-OH) in Patients with Amnestic Mild Cognitive Impairment (aMCI)

**DOI:** 10.3390/jcm14155481

**Published:** 2025-08-04

**Authors:** Seong Jae Kim, Jung Hie Lee, Jae-Won Jang, Minseo Choi, In Bum Suh

**Affiliations:** 1 Department of Psychiatry, College of Medicine, Chosun University, Gwangju 61452, Republic of Korea; 2 Department of Psychiatry, College of Medicine, Kangwon National University, Chuncheon 24341, Republic of Korea; 3 Department of Psychiatry, Gwanggyo Good Sleep Clinic, Suwon 16229, Republic of Korea; 4 Department of Neurology, College of Medicine, Kangwon National University, Chuncheon 24341, Republic of Korea; 5 Public Health Team, Jeju Institute of Public Health & Health Policy, Jeju 63243, Republic of Korea; 6 Department of Laboratory Medicine, College of Medicine, Kangwon National University, Chuncheon 24341, Republic of Korea

**Keywords:** amnestic mild cognitive impairment, rest–activity rhythm, 27-hydroxycholesterol, peripheral cholesterol oxygenation

## Abstract

**Background/Objectives**: Rest–activity rhythm (RAR) disturbances can contribute to aging and dementia via metabolic dysregulation. Hydroxycholesterol (OH) is thought to mediate the link between hypercholesterolemia and neurodegeneration. This study compared sleep and RAR parameters between amnestic mild cognitive impairment (aMCI) patients and normal controls (NCs), and examined their associations with plasma 27-OH levels, reflecting peripheral cholesterol metabolism. **Methods:** In total, 18 aMCI patients (76.6 ± 6.1 years) and 21 NCs (70.4 ± 6.7 years) underwent five-day actigraphy and dim light melatonin onset assessment. Plasma 27-OH levels were measured via high-performance liquid chromatography-mass spectrometry. Generalized linear models (GLMs) were used to analyze the relationships between sleep, RAR, and 27-OH levels. **Results**: The aMCI group had significantly lower 27-OH levels and 27-OH/total cholesterol ratios (*p* < 0.05). GLM revealed that longer sleep onset latency (SOL) was associated with higher 27-OH levels in aMCI, distinguishing them from NCs. Additionally, in aMCI, longer SOL, lower sleep efficiency (SE), and higher fragmentation index (FI) were associated with an increased 27-OH/total cholesterol ratio (*p* < 0.05). Higher relative amplitude of RAR was linked to lower 27-OH levels across groups (*p* < 0.01), but RAR parameters showed no significant association with the 27-OH/total cholesterol ratio. Sleep disturbances, including prolonged SOL, reduced SE, and increased FI, were associated with altered peripheral cholesterol oxygenation in aMCI. **Conclusions:** Greater RAR amplitude correlated with lower 27-OH levels, regardless of cognitive status. These findings suggest that peripheral cholesterol oxygenation in aMCI is related to both sleep disturbances and circadian rhythm dysregulation, highlighting their role in cholesterol metabolism and neurodegeneration.

## 1. Introduction

Sleep disturbances, including sleep fragmentation and excessive daytime sleepiness, are common in patients with Alzheimer’s disease (AD) [1] and have been implicated in the increased risk of cognitive impairment and the development and progression of AD. In addition to sleep disturbances, disruptions in circadian rhythms have emerged as a key factor in both cognitive and metabolic dysfunction. Circadian rhythms are essential for maintaining the homeostasis of behavioral patterns, such as sleep–wake cycles and eating behaviors, and for regulating various physiological functions, including glucose and lipid metabolism. Accordingly, circadian disruption has been associated with increased risk of metabolic syndromes, such as obesity and type 2 diabetes [2]. A previous study reported that older adults with metabolic syndrome exhibit a significantly more fragmented rest–activity rhythm (RAR) than those without it, suggesting that metabolic syndrome in older adults is associated with circadian rhythm disruptions [3].

These findings suggest a potential link between circadian rhythm disruption, metabolic dysfunction, and neurodegenerative disease. This raises interest in specific metabolic pathways that may bridge circadian disruption and neurodegeneration, most notably, lipid metabolism, which has been increasingly implicated in AD. Some epidemiological studies have reported associations between higher cholesterol levels and an increased prevalence of dementia [4,5]. However, other studies have reported conflicting results, showing no association or even inverse relationships, with lower cholesterol levels linked to increased dementia risk [6]. This discrepancy suggests that total cholesterol level alone may not adequately reflect the complex relationship between lipid metabolic dysfunction and AD pathology.

Although hypercholesterolemia is generally associated with an increased risk of neurodegenerative diseases, based on its presumed role in amyloid-β (Aβ) aggregation and neurofibrillary tangle formation, cholesterol itself cannot cross the blood–brain barrier (BBB) [7]. Therefore, peripheral cholesterol levels may have limited value in explaining central pathological changes in AD.

By contrast, oxysterols, oxidized derivatives of cholesterol, are able to cross the BBB and may serve as a functional link between peripheral cholesterol metabolism and brain pathology. Among these, 27-hydroxycholesterol (27-OH) and 24-hydroxycholesterol (24-OH) are two commonly studied forms. 27-OH is a circulating oxysterol mainly produced in the liver. High peripheral cholesterol levels are associated with elevated circulating 27-OH, which may enter the brain and contribute to AD-related neurodegeneration [6,8,9]. In addition to its absolute concentrations, the ratio of 27-OH to total cholesterol may reflect underlying changes in cholesterol turnover or its oxidative metabolism [10]. In contrast, 24-OH, which is synthesized exclusively in the brain, exits to the periphery and may serve as a marker of neuronal cholesterol metabolism [11]. A meta-analysis suggested that increased 24-OH levels in cerebrospinal fluid (CSF) are associated with AD pathology [12].

Despite this, the physiological role of oxysterols in neurodegeneration remains ambiguous. While many studies emphasize their deleterious effects, such as promotion of amyloidosis and cognitive decline, others suggest that elevated oxysterol levels may represent a compensatory response to cholesterol accumulation. For example, increased 27-OH levels have been observed in non-demented individuals as well as those with AD, and may indicate enhanced cholesterol turnover aimed at maintaining homeostasis [13,14].

Although growing evidence links sleep, circadian disruption, and cholesterol dysregulation to AD, few studies have explored their interconnections, particularly in the early stages of cognitive impairment. Amnestic mild cognitive impairment (aMCI) is generally considered a prodromal stage of AD. Thus, identifying metabolic or circadian markers associated with aMCI may offer valuable insight into early disease mechanisms and potential intervention targets [15].

Therefore, the aim of this study was to compare sleep characteristics and rest–activity rhythm between patients with aMCI and cognitively normal controls (NCs), and to examine how peripheral oxysterol levels, specifically 27-OH and 24-OH, relate to circadian and sleep parameters. We hypothesized that patients with aMCI would show altered sleep and RAR patterns compared to NCs, and that these disturbances would be associated with dysregulated cholesterol metabolism as reflected by peripheral oxysterol concentrations.

## 2. Materials and Methods

The study was approved by the Institutional Review Board of Kangwon National University Hospital (Chuncheon, Republic of Korea) and adhered to all relevant guidelines and regulations (approval no. KNUH-A-2020-06-005). Written informed consent was obtained from each participant and their legally authorized representatives before the commencement of this study. All procedures were performed in accordance with the principles of the Declaration of Helsinki.

### 2.1. Participants

Participants aged >50 years were recruited from the Memory Clinic at Kangwon National University Hospital, Chucheon, and two Public Centers for Dementia Care. The diagnosis of aMCI was established by a psychiatrist or neurologist according to Petersen’s criteria [16]. These criteria require the presence of subjective or objective memory impairment, the absence of dementia, and a score of −1.5 standard deviation below the age-, sex-, and education-adjusted normative value in at least one memory domain. In this study, the aMCI group comprised patients with isolated memory impairment (single-domain aMCI) and those with memory impairment plus one or more other cognitive dysfunctions (multidomain aMCI).

A screening interview was conducted to obtain the baseline information of the patient’s medical history, including any conditions or current medications. Participants (1) with current substance-related disorders, depressive disorders, or other psychiatric disorders diagnosed based on the Diagnostic and Statistical Manual of Mental Disorders, Fifth Edition; (2) with any conditions affecting the circadian rhythms (i.e., shift work and jet lag); (3) with current medical illness including liver cirrhosis, chronic pulmonary disease, cancer, uncontrolled diabetes, and uncontrolled hypertension; (4) with current use of any medications affecting sleep and the circadian rhythms (i.e., melatonin agents, Aspirin^®^, and sleeping pills); (5) who were suspected or diagnosed with primary sleep disorders were excluded. Participants taking lipid-lowering medications (e.g., statins) underwent a washout period of at least four times the drug’s half-life before blood sampling. Blood samples for lipid and oxysterol analyses were collected after ≥12 h of fasting [17].

Eighteen patients with aMCI (76.56 ± 6.1 years; M:F = 7:11) and 21 NCs (70.43 ± 6.97 years; M:F = 10:11) were ultimately selected for the study.

### 2.2. Procedures

#### 2.2.1. Neuropsychological Assessments

Neuropsychological assessments of eligible participants were conducted using the Korean version of the Consortium to Establish a Registry for Alzheimer’s Disease (CERAD-K) neuropsychological assessment battery [18]. This battery consists of 11 neuropsychological tests, including verbal fluency, the Modified Korean version of the Boston Naming Test, the Mini-Mental State Examination (MMSE) in the Korean version of the CERAD Assessment Packet (MMSE-KC), Word List Memory, Constructional Praxis, Word List Recall (WLR1), Word List Recognition (WLR2), Constructional Recall (CR), Trail Making Test-A (TMT-A), Trail Making Test-B (TMT-B), and the Stroop Color Word Test. Memory impairment was determined based on the WLM, WLR1, WLR2, and CR scores. This battery provides z-scores that are standardized based on age, sex, and educational level, which were used to evaluate cognitive performance in this study.

#### 2.2.2. Questionnaires

If the participants met the initial criteria, they completed additional questionnaires, including the Korean version of the Geriatric Depression Scale (GDS-K) [19], the Korean version of the Morningness–Eveningness Questionnaire (MEQ-K) [20], the Korean version of Epworth Sleepiness Scale (KESS) [21], Pittsburgh Sleep Quality Index (PSQI) [22], and Insomnia Severity Index (ISI) [23]. The 15-item Sleep Apnea Subscale, 12-item Narcolepsy Subscale, and 8-item Periodic Limb Movement Subscale (PLM) from the Sleep Disorders Questionnaire (SDQ) were used to determine the presence of primary sleep disorders [24].

#### 2.2.3. Actigraphy Monitoring

The participants were asked to wear an Actiwatch (Actiwatch 2; Philips Respironics, USA) on their non-dominant wrists, uncovered by clothing, at home for five consecutive days. Actigraphy was performed using a sleep diary. The actigraphy data were derived using Actiware-Sleep Software (version 6.0.2, Philips Respironics, Murrysville, PA, USA). A quality assessment of the actigraphy data was performed before analysis.

Sleep parameters were calculated based on the sleep period, defined as the time from lights off to lights on, according to their sleep diaries. The sleep parameters included time in bed (TIB), total sleep time (TST), sleep onset (SO), sleep onset latency (SOL), wake time after sleep onset (WASO), sleep efficiency (SE), and fragmentation index (FI).

The RAR was evaluated by nonparametric analyses using R statistical software(version 4.3.1, R Foundation for Statistical Computing, Vienna, Austria) and the “narACT” package [25]. The activity data from the actigraphs were aggregated into hourly bins for nonparametric analyses. Data with no activity signals for 1 h or longer were treated as missing. Missing hourly bins among the 24-hourly dataset were interpolated using the mean of the values obtained before and after the missing bin. If a missing periods lasted for more than 4 h, all data from that day were excluded from the analysis. The nonparametric variables, including interdaily stability (IS), intradaily variability (IV), and relative amplitude (RA), were derived. IS quantifies the invariability between days, while IV quantifies the frequency and extent of transitions between rest and activity. RA was calculated using data from the most active 10 h period (M10) and the least active 5 h period (L5) using the following formula: RA = (MI0 − L5)/(MI0 + L5). Among the 39 participants, two did not adhere to wearing the Actiwatch; therefore, data from only 37 participants were included in the RAR nonparametric analysis. Furthermore, data for all 5 days were used only for twenty-eight participants, while for the nine participants, some days could not be used for analysis due to missing activity data exceeding 4 h on certain days (2 participants missed 3 days, 4 participants missed 2 days, and 3 participants missed 1 day).

#### 2.2.4. Blood Sampling

Blood samples required for the analysis of lipid profiles, hydroxycholesterol levels, and ApoE genotyping were obtained during a clinic visit following the fifth day of actigraphy monitoring. The participants were required to fast for at least 12 h the night before the clinic visit until blood samples were drawn the next morning. Blood samples were centrifuged at 2000× *g* for 15 min at a temperature of 4 °C. After centrifugation, the serum was promptly transferred into cryovials and stored at −80 °C until analysis.

① Lipid profiles: The plasma concentrations of total cholesterol, triglycerides, low-density lipoprotein (LDL) cholesterol, and high-density lipoprotein (HDL) cholesterol were assayed using standard enzymatic procedures (CHOD-PAP method, Boehringer, Mannheim, Germany).

② Hydroxycholesterols: The analysis of OH, including 24-OH and 27-OH, was conducted using a triple quadrupole liquid chromatography-mass spectrometry (Finnigan TSQ Quantum Ultra EMR) system. High-performance liquid chromatography was performed using a receiver operating characteristic C18 column (3.0 × 150 mm, 5 µm, Restek) maintained at a column temperature of 30 °C, and mobile phases that consisted of 5 mM ammonium formate (solvent A) and 5 mM ammonium formate in methanol (solvent B). The mass spectrometer was equipped with an atmospheric pressure chemical ionization ion source in the positive mode, with a discharge current of 4, a vaporizer temperature of 300 °C, a sheath gas pressure of 43, an aux gas pressure of 11, and a capillary temperature of 280 °C.

**Figure 1:**
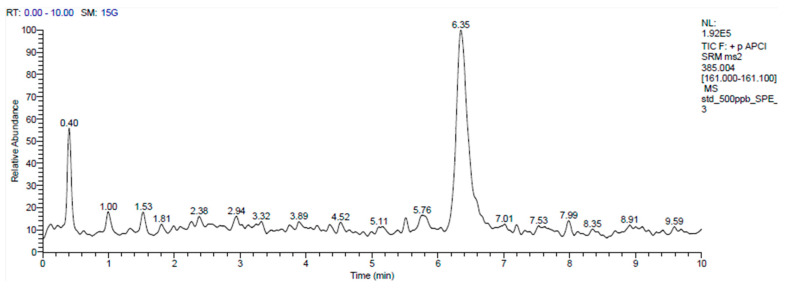
LC-MS/MS analysis was performed on a mixture containing 27-OH (500 ng, elution time 6.35 min). The 27-OH chromatogram shows signal intensity at various times. The main peak is located at approximately 6.35 min.

Sample preparation: Approximately 500 µL of serum were mixed with 5 mL of solution in a centrifuge tube. Then, 5 µL of internal standard (IS, 24-OH-d7, 10 ppm) were added to achieve a final concentration of 500 ppb. Alkaline hydrolysis was performed in 2 mL of 1 N ethanolic potassium hydroxide at 50 °C for 2 h. The hydrolyzed sample was subsequently neutralized to a pH of 7 with 75 μL of phosphoric acid. The mixture was centrifuged at 3000 rpm for 5 min after vortexing, and the supernatant was collected in another tube for solid-phase extraction. A hydrophilic–lipophilic balance (HLB) cartridge (3 cc, 60 mg, Waters Corporation, MA, USA) was placed in the manifold. The samples were loaded onto an HLB cartridge prepared by sequentially adding 1 mL of hexane:isopropanol (IPA) (1:1), methanol, and water. The cartridge was then washed with 4 mL of 75% methanol and dried for 1 min under a manifold. The analyte was eluted by gravity using 3 mL of hexane:IPA (1:1). The extracted samples were dried using a nitrogen concentrator (TurboVap LV, Caliper Life Sciences, Hopkinton, MA, USA) and subsequently evaporated using a speed vacuum concentrator (Savant SC210A, Thermo Fisher Scientific, Waltham, MA, USA). After dissolving the samples with 100 µL of methanol, the resulting solution was analyzed using mass spectrometry. Although both 24-OH and 27-OH were included in the initial analytical protocol, only 27-OH was reliably quantified. Due to technical limitations such as low endogenous concentration and matrix interference, 24-OH could not be consistently detected in the final analysis. The analysis only detected values for 27-OH (Figure 1).

③ ApoE genotype: Deoxyribonucleic acid (DNA) was extracted from frozen blood samples using the Gentra DNA Extraction Kit (protocol no. 00090, Gentra, Minneapolis, MN, USA). The extracted DNA was analyzed to determine the ApoE genotype using the polymerase chain reaction (PCR)-restriction fragment length polymorphism method (Bio-Core ApoE genotyping PCR, catalog no. 11081, Bio-Core Co., Ltd., Seoul, Republic of Korea). All procedures, including the interpretation of the ApoE genotype, were performed according to the instructions provided in the test kit. The ApoE alleles (ε2, ε3, and ε4) were identified using the methods described previously [26].

### 2.3. Statistical Analysis

To compare the demographic characteristics, the questionnaire and neurocognitive test scores, sleep parameters, RAR nonparametric variables, lipid profile, and 27-OH levels, and the presence or absence of ApoE ε4 between the aMCI and NC groups, the t-test or χ^2^ test was used as appropriate. The raw scores of the neurocognitive domains in the CERAD-K neuropsychological assessment battery were converted into z-scores, which represent the difference between each raw score and the mean of the corresponding subgroup, categorized by age, sex, and education level, divided by its standard deviation.

Generalized linear models (GLMs) were used to assess the main effects of sleep parameters or RAR nonparametric variables on 27-OH levels and their interactions with the group. The group (aMCI versus NC), sleep parameters (TIB, TST, SO, SOL, WASO, SE, and FI), and RAR nonparametric variables (IS, IV, and RA) were considered independent variables, while the 27-OH levels were considered dependent variables. The Shapiro–Wilk test for normality was applied to each dependent variable to determine the model type based on the data distribution. Additionally, the interactions between ApoE ε4 carrier and aMCI status on the 27-OH levels were measured using a two-way analysis of variance.

All statistical analyses were performed using the SPSS software package (version 18.0; SPSS Inc., Chicago, IL, USA). A two-sided *p*-value of less than 0.05 was considered significant.

## 3. Results

### 3.1. Demographic and Clinical Characteristics

As shown in Table 1, significant differences in age and GDS-K scores between the aMCI and NC groups were observed, as shown in Table 1 (*p* < 0.01). No significant differences were found in the MEQ-K, KESS, PSQI, or ISI scores between the two groups. The aMCI group had significantly lower scores on the PLM items of the SDQ subscales compared to the NC group (*p* < 0.05), as shown in Table 1.

### 3.2. Neurocognitive Functions

As shown in Table 2, the aMCI group had significantly lower scores than the NC group in all cognitive domains except for TMT-A and TMT-B (*p* < 0.05).

### 3.3. Nocturnal Sleep Parameters

As shown in Table 3, no significant differences were found between the aMCI and NC groups in TIB, TST, SOL, SE, WASO, or FI.

### 3.4. Nonparametric Variables of RAR

As shown in Table 4, no significant differences were noted in IV, IS, or RA between the MCI and NC groups.

### 3.5. Lipid Profiles and Hydroxycholesterol

As shown in Table 5, the levels of total cholesterol, triglycerides, LDL cholesterol, and HDL cholesterol did not differ significantly between the groups. However, the aMCI group exhibited significantly lower levels of 27-OH (*p* = 0.026) and a lower 27-OH to total cholesterol ratio (*p* = 0.025) compared to the NC group.

### 3.6. Effects of the Sleep Parameters on the 27-OH and 27-OH/Total Cholesterol Ratio

As shown in Table 6, GLMs revealed a significant interaction between group and SOL on 27-OH levels (*p* < 0.05), indicating that shorter sleep latency was associated with lower 27-OH levels in the aMCI group (β = 1.4), while it was correlated with higher levels in the NC group (β = −1.4).

As shown in Table 7, significant interaction effects were observed between the group and sleep parameters SOL, SE, and FI on the 27-OH/total cholesterol ratio. In the aMCI group, longer SOL, lower SE, and higher FI were associated with an increased ratio (β = 0.021, *p* = 0.029; β = −0.045, *p* = 0.033; β = 0.023, *p* = 0.036, respectively), unlike in the NC group.

### 3.7. Effects of the RAR Variables on the 27-OH and 27-OH/Total Cholesterol Ratio

As shown in Table 8, RA showed a significant main effect on 27-OH levels (β = −461.43, *p* < 0.01), indicating that higher RA was associated with lower 27-OH levels, regardless of group status.

However, as shown in Table 9, no significant interaction effects of IS, IV, or RA were observed on the 27-OH/total cholesterol ratio.

## 4. Discussion

In this study, the aMCI group exhibited more depressive symptoms, measured by GDS scores, compared with the NC group (Table 1). This finding aligns with those of previous studies, which reported that depression is the most common psychopathology associated with MCI [27]. Patients with aMCI subtype are more likely to have overt depressive symptoms than those with a non-amnestic subtype [27,28]. Additionally, our aMCI group exhibited deficits in multiple cognitive domains, including verbal ability, compared with the NC group (Table 2). Memory deficits and language impairments have been implicated as early signs of progression to AD [29] and have significant predictive value for conversion to dementia in aMCI [30].

Sleep disturbance is often considered a potential risk factor for cognitive decline, leading to the expectation that patients with MCI will experience more sleep disturbances than healthy controls [31]. Contrary to general expectations, our study did not find any significant differences in sleep parameters assessed by actigraphy between the aMCI and NC groups (Table 3). Only a few studies have used actigraphy to assess sleep quality in patients with MCI. In our earlier findings among patients with MCI, irrespective of the MCI subtype, no difference was found in sleep parameters, as measured by actigraphy, compared with the NC group [32]. Another study only showed a slightly lower SE in the MCI group (83.1%) compared with the NC group (85.7%), but did not find a significant difference in TST or WASO [33].

We observed no statistically significant differences in the RAR variables (interdaily stability (IS), intradaily variability (IV), and relative amplitude (RA)) between the aMCI and NC groups (Table 4). These findings are consistent with those of our previous study [32], which examined a small but heterogeneous MCI sample (n = 10) and similarly reported no significant group differences in RAR parameters compared to cognitively normal controls (n = 8). A previous study with 21 MCI patients and age-matched controls also found no significant differences in nonparametric RAR measures [34]. However, the lack of statistically significant RAR differences in our present study may reflect insufficient statistical power due to the small sample size, rather than the absence of circadian disruption in aMCI. This interpretation is further supported by a large-scale, community-based longitudinal study [35], which identified significant but modest reductions in RA among participants who developed MCI or Alzheimer’s disease over a 4-year follow-up. These findings suggest that subtle changes in circadian rhythms may be clinically meaningful but require large sample sizes and prospective designs to be reliably detected. In contrast, such differences may remain undetected in smaller, cross-sectional studies like ours, even when validated actigraphy-based assessments are used. Moreover, the relatively short actigraphy recording period (five days) in our study may have further limited sensitivity to detecting subtle differences in RAR patterns, particularly in older adults whose rest–activity rhythms may vary across days.

In our study, we examined whether disturbances in sleep, including sleep continuity and quality, as well as rest–activity rhythm (RAR), were associated with altered cholesterol metabolism, using generalized linear models (GLMs). Among the sleep-specific parameters, SOL was the only variable that showed a significant group-dependent association with 27-OH levels, notably differentiating the aMCI group from the NC group (Table 6). Specifically, in the aMCI group, longer SOL was associated with higher 27-OH levels (β = 2.83, *p* = 0.036), while this relationship was not significant in the NC group. In the same vein, SOL, sleep efficiency (SE), and fragmentation index (FI) also demonstrated significant associations with the 27-OH/total cholesterol ratio exclusively in the aMCI group, further underscoring a distinct sleep-related mechanism differentiating aMCI from NC (Table 7). A longer SOL was linked to an increased 27-OH/total cholesterol ratio (β = 0.021, *p* = 0.029), while lower SE (β = −0.045, *p* = 0.033) and higher FI (β = 0.023, *p* = 0.036) were associated with elevated ratios. These findings suggest an association between impaired sleep continuity and quality and altered cholesterol metabolism in patients with aMCI.

In contrast to sleep-specific variables, circadian rhythm parameters such as relative amplitude (RA) showed different associations. RA showed a significant negative association with 27-OH levels without significant between-group differences (Table 8). However, none of the circadian-related parameters were significantly associated with the 27-OH/total cholesterol ratio (Table 9). This indicates that while disrupted circadian rhythm may be associated with absolute 27-OH levels in both patients with aMCI and cognitively normal participants, their specific relationship with cholesterol metabolism, as reflected by the 27-OH/total cholesterol ratio, remains unclear. A previous study of 1137 older community-dwelling adults reported that more regular circadian rhythms, as assessed by the IS of RAR variables, were associated with a lower risk of metabolic syndrome (odds ratio = 0.69), including obesity, diabetes, hypertension, and dyslipidemia [36]. Nonetheless, our findings suggest potentially distinct physiological roles for circadian versus sleep mechanisms. Whether this relationship reflects a causal pathway, a compensatory response, or a shared underlying mechanism remains to be determined. Evidence from studies on mouse models indicates that 27-OH reduces cholesterol accumulation by lowering hepatic inflammation and improving liver metabolism [37], or by inhibiting cholesterol absorption [38], suggesting a beneficial effect of oxysterol on lipid homeostasis. One hypothesis is that elevated oxysterol levels may represent a compensatory response to sleep and circadian disturbances, potentially aiding in metabolic stability. While this remains speculative and requires longitudinal validation, further research is also needed to determine the underlying mechanisms and clinical implications of this relationship. Although exploratory GLM analyses revealed associations between certain circadian variables and 27-OH levels, no significant group-level differences in 27-OH were observed. This discrepancy underscores the need for cautious interpretation, as the findings may not reflect robust between-group effects. Furthermore, given the exploratory nature of the analyses and the absence of correction for multiple testing, the possibility of Type I errors cannot be excluded. These findings should therefore be considered hypothesis-generating rather than confirmatory.

Our study showed no significant differences in lipid profiles between the aMCI and NC groups (Table 5). This finding contrasts with those of previous studies, which reported that cognitively impaired individuals have higher cholesterol levels [39]. However, our findings are consistent with a long-term follow-up study indicating no notable cholesterol differences across MCI, AD, and healthy controls [40]. These conflicting outcomes may indicate that plasma cholesterol levels do not reliably reflect the lipid metabolism abnormalities associated with neurodegeneration. Notably, the aMCI group exhibited significantly lower 27-OH levels than the NC group. Additionally, the 27-OH to total cholesterol ratio was also significantly lower in the aMCI group compared to the NC group (*p* = 0.025). In contrast to our findings, Kommer et al. [6] reported that higher 27-OH/cholesterol ratios were associated with poorer cognitive performance in a community-based cohort of older adults. Furthermore, a meta-analysis by Wang et al. [13] found elevated peripheral 27-OH levels in patients with Alzheimer’s disease (AD) compared to healthy controls. Similarly, Björkhem et al. [8] also reported increased 27-OH concentrations in individuals with AD. Several factors may account for this discrepancy. Our study focused on a clinically well-defined population with aMCI, which represents an earlier and more specific stage of cognitive decline. In contrast, prior studies included broader or more heterogeneous populations, or individuals with more advanced neurodegenerative conditions. As such, our results may capture early disruptions in cholesterol metabolism that are distinct from the elevated 27-OH levels reported in Alzheimer’s disease or in more advanced stages of neurodegeneration. In particular, the reduced 27-OH levels and 27-OH/cholesterol ratios in our aMCI participants may reflect impaired peripheral cholesterol hydroxylation, possibly due to decreased activity of CYP27A1, the key enzyme mediating this process. This interpretation is supported by studies linking CYP27A1 polymorphisms to disrupted oxysterol metabolism in cognitively impaired individuals [41]. In support of our findings, several previous studies have also reported lower or unchanged 27-OH levels in cognitively impaired populations [14,17]. For instance, Kölsch et al. [14] found reduced 27-OH/cholesterol ratios in patients with Alzheimer’s disease, vascular dementia, and MCI compared to non-demented individuals. Similarly, Hughes et al. [17] reported that cholesterol metabolism markers, including oxysterols, were more strongly associated with cerebrovascular disease than with Alzheimer’s pathology. Although our study focused on aMCI, a clinically defined condition regarded as a prodromal stage of neurodegenerative disease, such as Alzheimer’s disease, the reduced 27-OH levels observed in this population may still reflect a convergence of underlying mechanisms. In particular, impaired neuronal integrity and downregulated CYP27A1 expression may affect the enzymatic conversion of cholesterol to 27-hydroxycholesterol [42]. Additionally, while not the primary focus in our cohort, subclinical cerebrovascular pathology, which is common in older adults, could also contribute indirectly by exacerbating oxidative stress or impairing metabolic regulation. These overlapping pathological processes may collectively explain the observed decline in 27-OH levels in individuals with aMCI.

Although our exploratory subgroup analysis showed numerically higher 27-OH levels and 27-OH/total cholesterol ratios in ApoE4 non-carriers compared to carriers, these differences did not reach statistical significance (*p* = 0.480 and *p* = 0.113, respectively). Given the small number of ApoE4 carriers (n = 8), the analysis was underpowered, and the results should be interpreted with caution. While ApoE plays a key role in cholesterol transport and has been implicated in cognitive decline [43], our findings are insufficient to determine whether ApoE genotype modulates 27-OH metabolism. Further studies with larger and genetically stratified cohorts are needed to clarify this relationship.

### Limitations

This study has several limitations. First, the relatively small sample size (n = 39), use of multiple exploratory statistical tests without formal correction, and single-center design with only Korean participants may limit statistical power, increase the risk of both Type I and Type II errors, and reduce the generalizability of the findings. Future studies with larger, multi-center, and ethnically diverse cohorts are needed to confirm and expand upon these results. Second, the aMCI group was significantly older than the NC group, which may have influenced the observed outcomes. Although age was statistically controlled in the main analyses, residual confounding effects cannot be entirely excluded. Future studies using age-matched groups are warranted. Third, although 24-hydroxycholesterol (24-OH) is a key brain-derived oxysterol, it could not be reliably quantified due to technical limitations and was thus excluded from the analysis. Future studies incorporating both 24-OH and 27-OH may provide a more comprehensive view of cholesterol metabolism in cognitive decline. Fourth, missing actigraphy data reduced the number of complete 5-day RAR datasets to 28 participants, potentially limiting the reliability and power of circadian rhythm analyses. Fifth, as polysomnography (PSG) was not performed, the presence of potential sleep disorders cannot be entirely ruled out and may have confounded the observed associations. In addition, the absence of neuroimaging data (e.g., MRI or PET) limited our ability to assess neurodegeneration or to directly link peripheral 27-OH levels to central nervous system pathology of Alzheimer’s disease.

## 5. Conclusions

Our study identified complex interactions between lipid metabolism, sleep, and circadian rhythms. These findings raise the possibility that oxysterols may play a dual role, potentially acting as a compensatory mechanism in response to sleep and RAR disturbances, and contributing to the maintenance of cognitive function in older adults. However, given the cross-sectional design of the study, causal inferences cannot be drawn, and the interpretations should be considered exploratory. Longitudinal studies are warranted to clarify these relationships.

## Figures and Tables

**Table 1:** Demographic and clinical characteristics in the NC and aMCI groups.

	NC (n = 21)	aMCI (n = 18)	*p*-Value
Age ^†^	70.43 ± 6.97	76.56 ±6.1 **	0.006
Sex (M:F) ^‡^	10:11	7:11	0.584
Education (year) ^†^	10.29 ± 2.99	7.78 ± 5.11	0.065
GDS-K	8.52 ± 4.52	13.33 ± 7.96 **	0.009
BMI	24.45 ± 2.83	23.57 ± 3.04	0.389
MEQ-K	61.95 ± 8.98	64.17 ± 6.82	0.564
KESS	7.33 ± 4.41	6.89 ± 4.48	0.496
PSQI	3.95 ± 2.8	3.28 ± 2.61	0.517
ISI	7.9 ± 5.53	6.78 ± 4.62	0.447
SDQ subscale			
NAR	15.57 ± 1.96	16.56 ± 4.57	0.564
SA	21.71 ± 5.67	21.56 ± 5.19	0.470
PLM	20.19 ± 4.99	17.67 ± 4.74 *	0.032
ApoE ε4 (non-carrier:carrier) ^‡^	15:5	12:4	1.00

*: *p* < 0.05; **: *p* < 0.01 (^†^ Independent *t*-test, ^‡^ X2 test or ANCOVA controlling for age).

**Table 2:** Neurocognitive test scores † in the NC and aMCI groups.

	**NC (n = 21)**	**aMCI (n = 18)**	***p*** **-Value**
VF	0.47 ± 1.13	−0.82 ± 0.54 **	0.001
BNT	0.65 ± 0.7	−0.33 ± 1.2 *	0.011
MMSE-KC	−0.09 ± 1.14	−0.95 ± 0.88 *	0.032
WLM	1.01 ± 1.07	−0.94 ± 0.78 **	0.000
CP	0.44 ± 0.64	−0.42 ± 1.46 *	0.027
WLR1	0.34 ± 0.78	−1.44 ± 0.83 **	0.000
WLR2	0.4 ± 0.53	−1.77 ± 1.49 **	0.000
CR	0.56 ± 1.03	−1.41 ± 0.62 **	0.000
TMT-A	0.6 ± 0.79	0.18 ± 0.68	0.136
TMT-B	0.5 ± 1.21	0.45 ± 0.84	0.699
SWCT	0.14 ± 0.81	−1.02 ± 0.8 *	0.002

*: *p* < 0.05; **: *p* < 0.01 (independent *t*-test). †: Given values are z-scores adjusted for age, sex, and education.

**Table 3:** Nocturnal sleep parameters in the NC and aMCI groups.

	NC (n = 20)	aMCI (n = 17)	*p*-Value
TIB (h)	8.62 ± 3.54	7.91 ± 1.52	0.659
TST (h)	6.08 ± 1.18	6.23 ± 1.33	0.840
SOL (min)	21.65 ± 16.27	20.52 ± 13.7	0.896
SE (%)	76.68 ± 7.2	77.79 ± 6.69	0.841
WASO (min)	67.56 ± 25.36	62.53 ± 25.69	0.530
FI	46.00 ± 14.65	45.25 ± 12.66	0.849

ANCOVA controlling for age.

**Table 4:** Nonparametric variables of RAR in the NC and aMCI groups.

	NC (n = 19)	aMCI (n = 15)	*p*-Value
IS	0.56 ± 0.16	0.61 ± 0.15	0.164
IV	0.75 ± 0.23	0.85 ± 0.33	0.496
RA	0.90 ± 0.92	0.89 ± 0.84	0.347

ANCOVA controlling for age.

**Table 5:** Lipid profiles and hydroxycholesterol in the NC and aMCI groups.

	NC (n = 21)	aMCI (n = 18)	*p*-Value
Lipid profiles			
Total cholesterol (mg/dL)	171.21 ± 45.59	180.25 ± 26.8	0.683
Triglyceride (mg/dL)	131.37 ± 72.38	133.75 ± 77.85	0.236
LDL-cholesterol (mg/dL)	96.74 ± 38.44	99.63 ± 23.85	0.744
HDL-cholesterol (mg/dL)	54.68 ± 15.42	51.63 ± 9.13	0.571
Hydroxycholesterol			
27-OH (ng/mL)	310.93 ± 69.73	267.71 ± 55.59 *	0.026
27-OH (ng/mL)/total cholesterol (mg/dL)	1.92 ± 0.54	1.51 ± 0.36 *	0.025

*: *p* < 0.05 (ANCOVA controlling for age).

**Table 6:** Main effects of the sleep parameters and their group interactions on the 27-OH.

	β	95% CI	*p*-Value
TST	8.75	−14.86–32.35	0.468
Group by TST	68.72	−139.23–276.67	0.282
SOL	−1.43	−3.06–0.20	0.082
Group by SOL	2.83 *	0.18–5.47	0.036
SE	0.71	−3.02–4.45	0.708
Group by SE	−4.3	−10.05–1.45	0.142
WASO	0.69	−0.39–1.76	0.210
Group by WASO	−0.12	−1.69–1.45	0.877
FI	−0.07	−1.88–1.74	0.940
Group by FI	2.3	−0.66–5.17	0.130

*: *p* < 0.05 (generalized linear model).

**Table 7:** Main effects of the sleep parameters and their group interactions on the 27-OH/total cholesterol ratio.

	β	95% CI	*p*-Value
TST	0.067	−0.180~0.120	0.542
Group by TST	−0.06	−0.11~−0.313	0.594
SOL	−0.016 **	−0.028~−0.005	0.006
Group by SOL	0.021 *	0.002~0.039	0.029
SE	0.016	−0.010~ 0.043	0.227
Group by SE	−0.045 *	−0.086~ −0.004	0.033
WASO	−0.001	−0.009~ 0.007	0.785
Group by WASO	0.010	−0.001~ 0.021	0.077
FI	−0.006	−0.019~ 0.007	0.355
Group by FI	0.023 *	0.001~ 0.044	0.036

*: *p* < 0.05; **: *p* < 0.01 (generalized linear model).

**Table 8:** Main effects of the RAR variables and their group interactions on the 27-OH.

	β	95% CI	*p*-Value
IS	−1.39	−178.25–175.48	0.988
Group by IS	−86.77	−366.19–192.65	0.543
IV	94.20	−26.91–215.30	0.127
Group by IV	−31.68	−186.30–122.94	0.688
RA	−461.43 *	−742.34–180.52	0.001
Group by RA	242.76	−218.94–704.45	0.303

*: *p* < 0.01 (generalized linear model).

**Table 9:** Main effects of the RAR variables and their group interactions on the 27-OH/total cholesterol ratio.

	β	95% CI	*p*-Value
IS	−0.326	−1.622~0.970	0.622
Group by IS	0.218	−1.830~2.266	0.835
IV	0.263	−0.660~ 1.186	0.577
Group by IV	0.233	−0.946~1.412	0.698
RA	−461.43	−3.446~1.357	0.394
Group by RA	−0.222	−4.169~3.725	0.912

Generalized linear model.

## Data Availability

The data supporting the findings of this study are available within the article.

## Abbreviations

The following abbreviations are used in this manuscript:

GDS-K	Korean version of the Geriatric Depression Scale
MEQ-K	Korean version of the Morningness-Eveningness Questionnaire
KESS	Korean version of the Epworth Sleepiness Scale
PSQI	Pittsburgh Sleep Quality Index
ISI	Insomnia Severity Index
CERAD-K	Korean version of the Consortium to Establish a Registry for Alzheimer’s Disease assessment battery
MMSE-KC	Mini-Mental State Examination in the Korean version of the CERAD Assessment Packet
VF	verbal fluency
BNT	Boston Naming Test
WLM	Word List Memory
WLR1	Word List Recall
WLR2	Word List Recognition
CP	constructional praxis
CR	constructional recall
TMT-A	Trail Making Test-A
TMT-B	Trail Making Test-B
SWCT	Stroop Word Color Test
TIB	time in bed
TST	total sleep time
SO	sleep onset
SOL	sleep onset latency
SE	sleep efficiency
WASO	wake after sleep onset
FI	fragmentation index
IS	interdaily stability
IV	intradaily variability
RA	relative amplitude
LDL	low-density lipoprotein
HDL	high-density lipoprotein
BMI	body mass index
ANCOVA	analysis of covariance
PCR	polymerase chain reaction
